# Procedures in child deaths in The Netherlands: a comparison with child death review

**DOI:** 10.1007/s10389-017-0800-9

**Published:** 2017-05-26

**Authors:** Sandra Gijzen, Jessica Petter, Monique P. L’Hoir, Magda M. Boere-Boonekamp, Ariana Need

**Affiliations:** 1 Department HTSR, IGS Institute for Innovation and Governance Studies, University of Twente, P.O. Box 217, 7500 AE Enschede, The Netherlands; 2 Division Nutrition and Health, Wageningen University, 6708 WE Wageningen, The Netherlands; 3 TNO Child Health, P.O. Box 2215, 2301 CE Leiden, The Netherlands; 4 Department Public Administration, IGS Institute for Innovation and Governance Studies, University of Twente, P.O. Box 217, 7500 AE Enschede, The Netherlands

**Keywords:** Child mortality, Child death review, Prevention, Implementation

## Abstract

**Aim:**

Child Death Review (CDR) is a method in which every child death is systematically and multidisciplinary examined to (1) improve death statistics, (2) identify factors that give direction for prevention, (3) translate the results into possible interventions, and (4) support families. The aim of this study was to determine to what extent procedures of organizations involved in the (health) care for children in The Netherlands cover these four objectives of CDR.

**Subject and methods:**

Organizations in the Eastern part of The Netherlands and Dutch umbrella organizations involved in child (health) care were asked to provide their protocols, guidelines or other working agreements that describe their activities and responsibilities in case of a child’s death. Eighteen documents and nine interview reports were made available. For the analyses we used scorecards for each CDR objective.

**Results:**

The procedures of Perined, the National Cot Death Study Group, Dutch Cot Death Foundation and Child Protection Service cover the largest part of the objectives of CDR. Organizations pay most attention to the translation of results into possible interventions. Family support gets the least attention in protocols, guidelines and other working agreements.

**Conclusion:**

Dutch organizations separately cover parts of CDR. When the procedures of organizations are combined, all CDR objectives are covered in the response to only specific groups of child deaths, i.e., perinatal deaths, Sudden Unexpected Deaths in Infants and fatal child abuse cases. Further research into the conditions that are needed for an optimal implementation of CDR in The Netherlands is necessary. This research should also evaluate the recently implemented NODOK procedure (Further Examination of the Causes of death in Children), directed to investigate unexplained deaths in minors 0–18 years old.

## Introduction

In The Netherlands, 992 children aged 0–19 (mortality rate 25.9/100,000) died in 2015, of which 84% were due to a natural cause (CBS 2015). Most children (56%) died under the age of 1 year mainly because of conditions originating in the perinatal period and congenital malformations, deformations and chromosomal abnormalities (CBS 2015). Almost half of the children aged 0–19 die in the hospital (CBS 2004). Although child mortality in The Netherlands has declined in the past decades (CBS 2015; Gijzen et al. 2013), each deceased child is one too many. Therefore, it is of great importance to *learn* from these deaths and to implement interventions preventing future deaths (Sidebotham and Pearson 2009).

In the USA, Canada, Australia, New Zealand and UK, the death of every child is examined in a systematic way by a multidisciplinary team. This method is called Child Death Review (CDR) (Durfee and Durfee, 2002; Durfee and Gellert, 1992; Sidebotham et al. 2008). A team of professionals collaborates according to a specific protocol. The kind of professionals who participate in the team differs among the countries where CDR is implemented (Fraser et al. 2014). The CDR objectives are to (1) improve the quality of the procedure with regard to the determination of the cause of death as well as the death statistics, (2) identify avoidable factors that give directions for prevention, (3) translate the results into possible interventions and (4) support the family (Covington et al. 2005; Cristian and Sege 2010; Ornstein et al. 2013; Sidebotham and Pearson 2009). Each country using the CDR has a different review process (Vincent 2014). However, all countries share the four objectives of CDR, which is considered to be the *gold standard* in the management of child deaths by the American Academy of Pediatrics (Cristian and Sege 2010).

It has been argued that there are many benefits of CDR and that a formal Child Death Review should be provided in all countries to understand how and why children die in order to prevent future child deaths (Fraser et al. 2014). In addition, according to the Convention on the Rights of the Child, every nation should take appropriate measures to diminish infant and child mortality (Unicef 1989). From this point of view, there seems to be a need to also implement CDR in The Netherlands. Parents in the first place, but also the Dutch government and local authorities are responsible for the wellbeing and safe development of every child. When a child dies, professionals from several organizations are involved. These professionals have different roles, tasks and responsibilities and approach the death of a child from different perspectives. Professionals have systematically reviewed cases of Sudden Unexpected Deaths in Infants (SUDI) since 1996 and perinatal deaths since 2009 in a multidisciplinary way in order to further prevent those deaths. In cases of unexplained death in minors efforts have been made, commissioned initially by the Ministry of Security and Justice, to develop the so-called NODO procedure (Further Examination of the Causes of Death; in Dutch: Nader Onderzoek DoodsOorzaak) starting from the first proposal by the consulting firm Van Montfoort in 2000. The necessary legislative changes were introduced in 2010 and 2012 (NVK/VWS 2016). The NODO procedure, requesting further examination of the child’s death in order to clarify the primary cause of death (Dutch-Government 2010; Dutch-Government 2012), was implemented in a national pilot test from 1 October 2012 to 31 December 2013. After the evaluation of the pilot period, the Ministry of Health, Welfare and Sport concluded that further examination into the causes of death should be organized regionally in a less extensive procedure. To achieve this, organizations involved in child deaths developed a multidisciplinary guideline that describes the procedure in case of unexplained death in minors (NVK/VWS 2016). This procedure, titled NODOK (Further Examination of the Causes of death in children, in Dutch Nader Onderzoek naar de DoodsOorzaak van Kinderen), has been in use since 1 August 2016 (FMG 2016) and implies a systematic investigation of the unexplained deaths by a multidisciplinary team, consisting of a pediatrician, forensic physician and pathologist, installed in six academic hospitals in The Netherlands.

In the eastern part of The Netherlands, a pilot implementation project of CDR was conducted from September 2009 to December 2013 [INTERREG Deutschland-Nederland (INTERREG Germany-The Netherlands)]. Within the framework of this pilot implementation, we performed a baseline measurement in which we inventoried how Dutch organizations involved in the (health) care for children responded to a child’s death in April 2011. We compared the characteristics of the organizations’ procedures with the objectives of CDR. In this context we answered the research question to what extent the existing procedures of organizations involved in the (health) care for children in The Netherlands cover the four CDR objectives in responding to a child’s death.

## Methods

### Study design

We used a qualitative, descriptive design to answer the abovementioned research question.

### Identification of stakeholders

An inventory of organizations that are involved in the (health) care for children in the eastern part of The Netherlands was made on the basis of the type of organizations in the UK that are working with children and are responsible for their safety and development (UK-Government 2013). The identified 22 Dutch stakeholder organizations are outlined in Table 1, of which one is the hospital (department of pediatrics). Eight hospitals are identified in the pilot region, including one top clinical hospital with a neonatal intensive care unit. Six stakeholders are only organized on a national level (Table 1). In case of a child’s death (part of) the listed organizations in Table 1 use a protocol, guideline or other type of working agreement.

**Table 1:** Overview of Dutch organizations involved in a child’s death, their protocols, guidelines and other working agreements included for analysis. In brackets the number of professionals who were approached to provide documents

Organization	Title of document available for analysis	Description of the tasks/activities
1. Hospital department of pediatrics [10]	a. Dutch Association for Pediatrics-Action protocol after cot death	Procedures are aimed at determining the cause of death and avoiding false suspicion of parents
	b. Death of a child (at emergency dept.)	Attention is paid to nearness to the dying child, spiritual care and aftercare for parents. Supporting parents is most important in this protocol
	c. Emergency baptism at an infant’s death	This protocol provides for an emergency baptism when a child is dying and the parents want their child baptized. Parents’ wishes are central
	d. Deceasing or dying	The aim is taking leave of the dying one and providing spiritual care
	e. Procedures for external cause of death	A few points of interests are briefly described, e.g., the execution of the autopsy and informing the family. It is hard to classify this protocol
2. General practice [1]	Dutch Association for Pediatrics—Action protocol after cot death (*same as organization 1*)	Procedures are aimed at determining the cause of death and avoiding false suspicion of parents
3. Forensic medical service-part of the Municipal Health Service [2]	a. Work instruction ‘reporting deceased minors’	According to a flowchart the municipal forensic physician draws a conclusion about the manner and cause of death
	b. Guideline forensic postmortem examination	The protocol describes the responsibilities of the municipal forensic physician, what to determine (e.g., cause of death), who to inform about the death and how to report
4. Ambulance service [2]	National protocol ambulance care	The main aim of this guideline is providing for acute assistance. Some attention is paid to (determining) SUDI and to the grieving process
5. Preventive child healthcare [1]	Guideline counseling families in child death	When a child dies Preventive Child Healthcare contacts the parents to console them and to inform them about aftercare regarding the grieving process
6. Municipal health services [1]	a. Guideline counseling families in child death (*same as organization 5*)	When a child dies Preventive Child Healthcare contacts the parents to console them and inform them about aftercare regarding the grieving process
	b. Protocol large-scale sexual abuse	This protocol could be used to prevent social tumult in the context of child mortality. Relief and assistance are part of it
7. Hospital social work [1]	Interview report	A memorial day for deceased children is organized in the hospital without guidelines, so no protocol could be analyzed
8. General social work [1]	No usable protocols	The protocol retrieved was not aimed at child mortality
9. Mental Health Trust [3]	a. Suicide and external cause of death	The main aim is informing the right professionals and organizations and reporting about the death. None of the four objectives is central; therefore, this protocol has not been classified
	b. External cause of death in admitted patient inside of the clinic	Responsibilities of the professionals involved in the context of determining the cause of death and grief counseling are described
	c. External cause of death in admitted patient outside of the clinic	Responsibilities of the professionals involved in the context of determining the cause of death and grief counseling are described
	d. External cause of death in ambulatory patient outside of the clinic	Administrative tasks of the professionals involved aimed at determining the cause of death are central
10. MEE [1]	Interview report	This organization does not use protocols in case of child death
11. Child Welfare Agency [1]	Guidelines death of a juvenile client	This protocol is a practical description of informing the right professionals and organizations. Some attention is paid to supporting the professionals involved and the family
12. Child Protection Service [1]	Interview report	The protocol retrieved was not aimed at child mortality
13. Police [1]	Interview report	Procedures are performed to determine cause of death
14. Public prosecutor [1]	Interview report	Procedures are performed to determine cause of death
15. School/daycare/playgroup [4]	Protocol in case of death	This protocol is a general guideline how to deal with practical aspects of providing information, organizational adjustments and grief counseling in case of a child’s death
16. Perined^a^ [1]	Local audit	Professionals analyze the provided care in a structured way to improve the quality of care. Getting insight in avoidable factors in perinatal deaths is important
17. National Cot Death Study Group^a^ [1]	Dutch Cot Death Foundation	Professionals analyze the provided care in a structured way to investigate whether SIDS was the cause of death. Preventing SIDS and informing and advising parents are the main goals
18. Dutch Cot Death Foundation^a^ [1]	Interview report	This organization delivers evidence-based information for professionals and parents by means of a website
19. Association for Parents of a Deceased Child^a^ [2]	No protocols	No protocols retrieved because of no response
20. Dutch Safety First Association [1]	Interview report	This organization focuses on developing interventions in the context of child mortality
21. Consumer Safety Institute^a^ [1]	Interview report	This organization focuses on developing interventions in the context of child mortality
22. Dutch Safety Board^a^ [1]	Interview report	This organization focuses on developing interventions in the context of child mortality

^a^Organized on a national level

### Identification of CDR characteristics

We used the UK CDR method, as described in the document ‘*Working Together to Safeguard Children*’ (UK-Government 2013), to identify a list of objectives to analyze in our study. CDR in the UK is a standardized process that is described clearly and in detail, and it includes all child deaths (Fraser et al. 2014). It consists of two interrelated parts: (1) the Rapid Response (RR), undertaken by a special team immediately after a sudden and unexpected death of a child, and (2) the Child Death Overview (CDO) undertaken by a panel, a few months after a child death, including the RR cases. The RR team is directed at determining the cause of death, identifying any contributory factors and ensuring ongoing support of the family (Sidebotham and Pearson 2009). CDO panels’ main targets are systematic analysis of the information provided by the professionals who were involved before and around the time of death in order to identify modifiable factors, making recommendations for prevention and signaling patterns or trends in child deaths.

The different characteristics mentioned in the description of the RR and CDO (UK-Government 2013) were used as criteria to determine the extent to which the procedures of Dutch organizations cover the four CDR objectives. The characteristics of RR and CDO were identified by the second author and arranged according to the four CDR objectives. In the final list of characteristics (Table 2a and b), the number of characteristics varies by CDR objective. The whole procedure of making the list of characteristics was checked by the first and fourth author independent of each other, and differences were discussed until consensus was reached. Prerequisites like working agreements directed at communication were not included in the set of characteristics.

**Table 2:** Characteristics of the Rapid Response (RR) and Child Death Overview (CDO), arranged according to the Child Death Review objective, and organizations that have these characteristics as a major or minor focus. (a,b,c,d) refer to the documents available for analysis mentioned in Table 1

Organizations with procedures with			
		Major focus (+ in the appendix)	Minor focus(± in the appendix)
A			
Objective ‘Improve the quality of the procedure with regard to the determination of the cause of death as well as the death statistics’			
RR	Rapid response actions exist in cases of unexplained death	Dept. of Pediatrics (a), GP, Forensic Med. Service (a), Cot Death Found.	Child Welfare Agency, Child Protection Service, Publ. Prosecutor, National Cot Death Study Group
	It has been defined who will lead the investigation to determine the cause of death	Forensic Med. Service (a), Mental Health Services (b), Mental Health Trust (b,c,d), Child Protection Service, Police, Publ. Prosecutor, Perined, National Cot Death Study Group, Cot Death Found.	Dept. of Pediatrics (a), GP, Social Work in hosp.
	It has been defined which professionals have to be involved in the investigation to determine the cause of death	Dept. of Pediatrics (a), GP, Forensic Med. Service (a), Mental Health Trust (b,c,d), Child Protection Service, Police, Publ. Prosecutor, Perined, Cot Death Found.	Forensic Med. Service (b), Ambulance Service, Mental Health Trust (a), Child Welfare Agency, Cot Death Comm.
	It has been defined what has to be investigated. This includes: data collection from relevant institutions and professionals, postmortem investigation and investigation at the place of death and circumstances of the death	Dept. of Pediatrics (a), GP, National Cot Death Study Group	Dept. of Pediatrics (e), Forensic Med. Service (a,b), Mental Health Trust (b,c,d), Child Protection Service, Police, Publ. Prosecutor
	Results are collected and represented according to national criteria	Dept. of Pediatrics (a), GP, Child Protection Service, Police, Publ. Prosecutor, Perined	Forensic Med. Service (a), Mental Health Trust (b,c,d), Cot Death Found.
	It has been defined how the relationship between physicians and the forensic physician could be constituted	Forensic Med. Service (a)	Cot Death Found.
	It has been defined how often and when the involved professionals have to discuss the results of the investigation to determine the cause of death	Forensic Med. Service (a), Police, Publ. Prosecutor	Dept. of Pediatrics (a), GP, Forensic Med. Service (b), Munic. Health Services (b), National Cot Death Study Group
	Relevant institutions and professionals, such as school and GP, are consulted to get relevant information about the possible cause of death	Child Protection Service, Police, Publ. Prosecutor, National Cot Death Study Group	Forensic Med. Service (b), Munic. Health Services (b)
CDO	The results of the review are passed on to a national institution	Dept. of Pediatrics (a), GP, Mental Health Trust (b,c,d), Perined, National Cot Death Study Group	Child Protection Service, Publ. Prosecutor, Cot Death Found.
	A format to get specific data about a particular cause of death is used	Dept. of Pediatrics (a), GP, National Cot Death Study Group	Perined, Cot Death Found.
	The actions of professionals involved in determining the cause of death are analyzed	-	-
	Feedback is given to professionals on their actions in determining the cause of death	-	-
	New relevant information regarding the cause of death and factors contributing to the death, which is obtained in the long run, is provided to all professionals involved in the death	Child Protection Service, Publ. Prosecutor, Perined	Police, Cot Death Found.
Objective ‘Identify avoidable factors that give directions for prevention’			
RR	Relevant institutions and professionals, such as school and GP, are consulted to get more information about the child, his/her social circumstances and environment in the context of avoidable factors of child mortality	Child Protection Service, Cot Death Found., Safety Board	Forensic Med. Service (b), Munic. Health Services (b), Publ. Prosecutor, Perined, National Cot Death Study Group, Consumer Safety Inst.
	During data collection from relevant institutions and professionals, postmortem examination and investigation at the place of death and circumstances of the death, attention is paid to (new) avoidable factors of child mortality	Dept. of Pediatrics (a), GP, Child Protection Service, Cot Death Found.	Publ. Prosecutor, National Cot Death Study Group
CDO	Avoidable factors of child mortality and lessons learned are identified	Dept. of Pediatrics (a), GP, Child Protection Service, Perined, National Cot Death Study Group, Cot Death Found.	Mental Health Trust (a), Police, Publ. Prosecutor, Safety First Assoc., Consumer Safety Inst., Safety Board
	A distinction is made in factors intrinsic to the child, family and environmental factors, parenting capacity, and service provision	-	Child Protection Service, Perined, Consumer Safety Inst., Safety Board
	Professionals involved work together with regional and national institutions to identify lessons learned	Dept. of Pediatrics (a), GP, Child Protection Service, Perined, National Cot Death Study Group, Cot Death Found.	Social Work in Hosp., Publ. Prosecutor, Safety First Assoc., Consumer Safety Inst., Safety Board
	After identifying avoidable factors of child mortality, the extent of the problem is determined and (groups of) people most affected by the problem are sorted out	Perined, National Cot Death Study Group, Cot Death Found., Safety Board	Child Protection Service, Publ. Prosecutor, Safety First Assoc., Consumer Safety Inst.
B			
Objective ‘Translate the results into possible interventions’			
RR	Information relevant for immediate prevention (e.g., protection of other children in the family) is discussed by the rapid response team	Munic.Health Services (b), Mental Health Trust (b,c), Child Welfare Agency, Child Protection Service	Ambulance Service, Police
	It has been defined which immediate preventive measures have to be taken, when necessary	Mental Health Trust (b,c), Child Protection Service	Child Welfare Agency
CDO	Research ends with a discussion how such a death can be avoided in the future	Perined, National Cot Death Study Group, Cot Death Found.	Mental Health Trust (b,c), Child Protection Service, Safety Board
	Recommendations, actions to be performed and lessons learned are passed on to relevant authorities or individuals	Mental Health Trust (b,c), Child Protection Service, Perined, National Cot Death Study Group, Cot Death Found., Safety First Found., Consumer Safety Inst., Safety Board	Munic. Health Services (b), Mental Health Trust (a,d), Publ. Prosecutor
	Recommendations, actions to be performed and lessons learned are passed on to governmental institutions to improve Publ. health	National Cot Death Study Group, Cot Death Found., Safety First Found., Consumer Safety Inst., Safety Board	Child Protection Service, Publ. Prosecutor, Perined
	It has been defined who is responsible for (taking care of) carrying out the improvements	Perined, Safety First Found., Consumer Safety Inst., Safety Board	Mental Health Trust (b,c)
Objective ‘Support to the family’			
RR	The potential needs of relatives are identified	Dept. of Pediatrics (b,d), Preventive Child Healthcare, Munic. Health Services (a,b,), Social Work in Hosp., Child Welfare Agency, School	Dept. of Pediatrics (a,c), GP, Mental Health Trust (b,c), Publ. Prosecutor, National Cot Death Study Group, Cot Death Found.
	When a child died in the hospital, parents are supported by a designated professional of the hospital	Dept. of Pediatrics (b), social work in hosp.	Dept. of Pediatrics (d), Munic. Health Services (b)
	When conditions permit, parents get the opportunity to be alone with their deceased child to take leave of their child	Dept. of Pediatrics (b,d)	
	Parents are informed about up-to-date findings of the research, unless this obstructs the research	Dept. of Pediatrics (a), GP, Munic. Health Services (b), Police	Dept. of Pediatrics (d), Mental Health Trust (b,c)
	It has been defined how to act when parents and the deceased child do not live in the same country	-	-
	After completion of the rapid response, further (psychological) assistance is rendered to the relatives	Dept. of Pediatrics (b), Preventive Child Healthcare, Munic. Health Services (a,b), Social Work in Hosp., Mental Health Trust (a,b,c,d), Cot Death Found.	Child Welfare Agency, School
CDO	The actions of professionals in supporting grief counseling to relatives are analyzed	Dept. of Pediatrics (b), Munic. Health Services (b)	Social Work in Hosp., Mental Health Trust (a)
	Relatives are kept in touch in the long run, whereby feedback is given on research of (factors contributed to) the death and grief counseling	Dept. of Pediatrics (b), Munic. Health Services (b), National Cot Death Study Group, Cot Death Found.	Dept. of Pediatrics (a), GP, Preventive Child Healthcare, Munic. Health Services (b), Social Work in Hosp., Child Protection Service, School, Perined
	The given support to relatives is monitored	Preventive Child Healthcare, Munic. Health Services (a,b), social work in hosp.	Dept. of Pediatrics (c), Mental Health Trust (a), Cot Death Found.

*Dept*. department, *GP* general practitioner, *Med*. medical, *Found*. foundation, *Publ*. public, *Hosp*. hospital, Munic. municipal, *Inst*. institute, *Assoc*. association

### Data collection

In April 2011 all inventoried organizations were asked to provide information on procedures, laid down in established protocols, guidelines or other working agreements (referred to below as ‘guidelines’) that describe their responsibilities and activities in case of a child’s death. If written guidelines were not available, information was obtained by means of semi-structured interviews with professionals as representatives of their organizations. These interviews were written out. Main characteristics of the procedures concerning the responsibilities and activities of that organization in responding to child deaths were identified. Subsequently, it was determined what CDR objective(s) correspond(s) with regard to these characteristics.

One out of 22 organizations (Table 1) did not respond to our request (parents’ association). Of the remaining 21 organizations, 12 provided a total of 18 guidelines that were relevant for answering our research question.

Of the 21 organizations, 9 did not have any written guideline that describes how to act in case of a child’s death. Representatives of those nine organizations (Hospital Social Work; MEE, an organization that provides support to people with intellectual disabilities or chronic illness; Child Protection Service; Police; Public Prosecutor; Dutch Cot Death Foundation; Dutch Safety First Association; Dutch Consumer and Safety Institute; and Dutch Safety Board) were asked for an interview. The website of the Dutch Cot Death Foundation has been part of the Netherlands Centre Youth Health (NCJ) website since April 2015. Eighteen written guidelines and nine interview reports were available for analysis (Table 1).

We did not include the concept NODO procedure as it had not yet been established in April 2011. However, the work instruction “Reporting deceased minors” (valid and mandatory from 1 January 2010; developed for the purpose of the consultation of a municipal coroner) was one of the 18 written guidelines.

### Data analysis

To measure the extent to which the procedures of the abovementioned organizations cover the four CDR objectives, scorecards were used with the characteristics arranged by the CDR objective. For each of the 18 retrieved written guidelines and 9 interview reports a scorecard was filled in. The question whether the description of responsibilities and activities in the guidelines and interview reports corresponded with the characteristics of CDR on the scorecard could be answered with ‘yes,’ ‘to a limited extent’ or ‘no.’ In case of uncertainty the guideline or interview report was scored again by the second author and discussed with the third author after which a definitive decision was made. Finally, for each of the guidelines and interview reports the second author completed the scorecards.

## Results

The extent to which the procedures of organizations involved in the (health) care for children in The Netherlands cover the four CDR objectives is shown in Table 2a, b and Appendix 1, 2, 3 and 4. Below, for each of the CDR objectives, we summarize the findings.

### ‘Improve the quality of the procedure of determining the cause of death as well as the quality of the causes of death statistics’

The CDR objective directed at the improvement of the quality of the procedure with regard to the determination of the cause of death as well as the death statistics is mainly found in the ‘Action protocol after cot death’ of the Dutch Association for Pediatrics and the procedures of the Public Prosecutor, the Child Protection Service and the National Cot Death Study Group (Table 2a and Appendix 1).

Half of the participating organizations describe in their procedures which professionals have to be involved in the investigation in determining the cause of death shortly after the death of a child. Only two organizations, the Forensic Medical Service and the National Cot Death Study Group, pay (some) attention to defining how the collaboration between physicians and the municipal forensic physician could be constituted (Table 2a and Appendix 1).

Eight organizations describe in their procedures that results of the review need to be passed on to a national institution a few months after the death of a child. No organization focuses in their procedures on the need to analyze the actions of professionals in determining the cause of death and to provide feedback on this to improve the quality of the procedure with regard to the determination of the cause of death (Table 2a and Appendix 1).

### ‘Identify avoidable factors that give directions for prevention’

In general, the CDR objective directed at the identification of avoidable factors that give directions for prevention is most recognizable in the procedure of the Child Protection Service, Perined, the National Cot Death Study Group and the Dutch Cot Death Foundation (Table 2a and Appendix 2).

Only three organizations specifically describe in their procedures that relevant institutions and professionals should be consulted in order to register possible avoidable factors shortly after the death of a child. Also, four organizations have their major focus on recording (new) avoidable factors of child deaths during the investigation (Table 2a and Appendix 2).

Six organizations have a major focus on the identification of avoidable factors and learned lessons as well as on working together with regional and national institutes to identify learned lessons a few months after the death of a child. None of the organizations has a major focus in their procedures on the categorization in factors intrinsic to the child, the family and environment, the parenting skills and service provision. Of the four organizations that have a minor focus in their procedures on this characteristic, only the Consumer Safety Institute distinguishes between behavioral, product and physical factors (Table 2a and Appendix 2).

### ‘Translate the results into possible interventions’

The CDR objective directed at the translation of identified factors into possible interventions is mainly displayed in the procedures of the institutes for mental health care directed at external causes of death in- and outside the clinic and the procedure of the Child Protection Service (Table 2b and Appendix 3).

In the procedures of four organizations specific attention is paid to discussing information for immediate prevention shortly after the death of a child. Only three organizations have defined in their procedures which preventive actions should be taken (Table 2b and Appendix 3).

Eight organizations particularly focus in their procedures on the aspect of informing relevant authorities and individuals a few months after the death of a child about the recommendations, actions to be performed and lessons learned. In the procedures of only three organizations it is specifically described that an investigation ends with a discussion of how to prevent such a death in the future (Table 2b and Appendix 3).

### ‘Support of the family’

The CDR objective directed at the support of the family is mainly included in the procedures of the Department of Pediatrics described in ‘Death of a child,’ of the Hospital Social Work, and of the Municipal Health Services, directed at prevention of social anxiety in serious traumatic incidents, for example, in case of child abuse and child deaths (Table 2b and Appendix 4).

Half of the participating organizations pay attention to the potential needs of relatives shortly after the death of a child, for example, needs concerning washing and dressing the deceased child and farewell rituals. No organization, except the department of pediatrics in the hospital, describes that parents get the opportunity to be alone with their deceased child to take leave of their child. In addition to this, no organization describes in their procedures how to act in the rare situation that the parents and the deceased child do not live in the same country (Table 2b and Appendix 4).

Almost half of the participating organizations describe in their procedures the follow-up of relatives a few months after the death of a child, where feedback is given about the circumstances of and factors that contributed to the death and grief counseling is provided. The analysis of the actions of professionals in supporting grief counseling to relatives is described in the procedures of only four organizations (Table 2b and Appendix 4).

## Discussion

Quite a few organizations are involved in child deaths in The Netherlands. The procedures of these organizations, laid down in protocols, guidelines and working agreements, obtained in April 2011, were systematically compared to the objectives of CDR. In the analysis it was determined to what extent the procedures cover the four objectives of CDR used in the UK, namely the (1) improvement of the quality of the procedure with regard to the determination of the cause of death as well as the causes of death statistics, (2) identification of avoidable factors that give directions for prevention, (3) translation of results into possible interventions and (4) support of the family.

When all procedures of Dutch organizations in responding to child deaths are combined, the four CDR objectives are largely covered in the response of these organizations, but only for specific groups of child deaths, namely for perinatal deaths (Perined), SUDI cases (National Cot Death Study Group and Dutch Cot Death Foundation) and fatal child abuse cases (Child Protection Service). It is indisputable that all organizations (should) devote attention to support involved relatives.

These results imply that the different procedures are fragmented in relation to the objectives of CDR and that not all groups of child deaths are covered, such as natural causes of child death other than perinatal deaths and SUDI and death due to intentional self-harm. We consider the insufficient coverage as a shortcoming, because it provides us an incomplete overview of avoidable factors in child deaths that hinders targeted preventive measures. With regard to fragmentation this is not necessarily disadvantageous as long as organizations are aware of their tasks and the tasks of other organizations in case of a child’s death and communicate and share information with each other (Durfee and Parra, 2009; Durfee and Gellert, 1992; UK-Government 2013). Reviews on child’s death and serious injury in different countries have stressed the importance of inter-agency working (Axford and Bullock, 2005). To take adequate actions to prevent a child’s death and to support the family, clear local arrangements for collaboration between organizations are needed.

### Strengths and weaknesses of this study

One of the strengths of this study is the broad scope that is used to identify the organizations and to analyze their procedures. Another strength is the high response rate of the organizations that have been approached. Only one organization, the parents’ association, did not react to our request to participate in this study. Although all hospitals in our pilot region have been approached and gave insight in their procedures, the procedures of the academic hospitals located outside our study region were not obtained. Therefore, some caution is required in the interpretation of the results as some of the children die in an academic hospital. Apart from this limitation, the quantity of retrieved procedures provides us an almost complete overview of the procedures in responding to child deaths in the Eastern part of The Netherlands and of some organizations involved at a national level in April 2011. However, the NODOK procedure, which has been in use since August 2016, was not evaluated in our study. The systematic analysis of cases of unexplained death in children up to 18 years old according to this NODOK procedure undoubtedly includes several aspects of the CDR objectives.

A weakness in this study is the fact that we did not examine whether and to what extent the organizations actually *act* in case of a child’s death according to these procedures. Professionals within these organizations may provide other care than defined. We also did not examine to what extent organizations have a multidisciplinary case discussion within their own organization after a child has died. Further research could give insight into the adherence to protocols, guidelines or other working agreements by professionals.

## Conclusions

Whereas CDR examines all child deaths, the procedures of the organizations in this study that cover parts of the four CDR objectives focus on a *particular part* of child mortality only. Consequently, a complete overview of avoidable factors that give directions for prevention of child deaths is lacking. Another conclusion is that support of the family should be more systematically included in the procedures of organizations.

Further research into the conditions that are needed for an optimal implementation of CDR in The Netherlands is necessary. If the responsibilities and activities were better coordinated among organizations involved, the four objectives of CDR could be better achieved in the majority of (natural) child deaths. CDR might then only be indicated for particular groups of child deaths, e.g., in unexpected, unexplained child deaths, to achieve its objectives. The recently implemented NODOK procedure may provide this systematic approach in this particular group of children.

## Appendix 1

**Table 3:** Extent to which procedures of Dutch organizations covered the CDR objective ‘*Improve the quality of the procedure with regard to the determination of the cause of death as well as the death statistics’* (yes +; to a limited extent = ±; no = −)

Organization/ professional	Title of document available for analysis	RR1.1	RR1.2	RR1.3	RR1.4	RR1.5	RR1.6	RR1.7	RR1.8	CDO1.1	CDO1.2	CDO1.3	CDO1.4	CDO1.5
Department of Pediatrics and GP	Dutch Association for Pediatrics-Action protocol after cot death	+	±	+	+	+	−	±	−	+	+	−	−	−
	Death of a Child	−	−	−	−	−	−	−	−	−	−	−	−	−
	Emergency Baptism	−	−	−	−	−	−	−	−	−	−	−	−	−
	Deceasing or Dying	−	−	−	−	−	−	−	−	−	−	−	−	−
	Procedures in External Cause of Death	−	−	−	±	−	−	−	−	−	−	−	−	−
Forensic Medical Service	Work Instruction ‘Reporting Deceased Minors’	+	+	+	±	±	+	+	−	−	−	−	−	−
	Guideline Forensic Postmortem Examination	−	−	±	±	−	−	±	±	−	−	−	−	−
Ambulance Service	National Protocol Ambulance Care	−	−	±	−	−	−	−	−	−	−	−	−	−
Preventive Child Healthcare/Municipal Health Services	Guideline Counseling Families in Child Death	−	−	−	−	−	−	−	−	−	−	−	−	−
	Protocol Large scale Sexual Abuse	−	+	−	−	−	±		±	−	−	−	−	−
Hospital Social worker	Interview report	−	±	−	−	−	−	−	−	−	−	−	−	−
Mental health trust	Suicide and External Cause of Death	−	−	±	−	−	−	−	−	−	−	−	−	−
	External Cause of Death Inside of the Clinic	−	+	+	±	±	−	−	−	+	−	−	−	−
	External Cause of Death outside of the Clinic	−	+	+	±	±	−	−	−	+	−	−	−	−
	External Cause of Death in Ambulatory Patient Outside of the Clinic	−	+	+	±	±	−	−	−	+	−	−	−	−
MEE	Interview report	−	−	−	−	−	−	−	−	−	−	−	−	−
Child Welfare Agency	Guidelines Death of a Juvenile Client	±	−	±	−	−	−	−	−	−	−	−	−	−
Child Protection Service	Interview report	±	+	+	±	+	−	−	+	±	−	−	−	+
Police	Interview report	−	+	+	±	+	−	+	+	−	−	−	−	±
Public Prosecutor	Interview report	±	+	+	±	+	−	+	+	±	−	−	−	+
School	Protocol in Case of Death	−	−	−	−	−	−	−	−	−	−	−	−	−
Perined	Perined	−	+	+	−	+	−	−	−	+	±	−	−	+
National Cot Death Study Group	Dutch Cot Death Foundation	±	+	±	+	−	−	±	+	+	+	−	−	−
Dutch Cot Death Foundation	Interview report	+	+	+	−	±	±	−	−	±	±	−	−	±
Dutch Safety First Association	Interview report	−	−	−	−	−	−	−	−	−	−	−	−	−
Consumer Safety Institute	Interview report	−	−	−	−	−	−	−	−	−	−	−	−	−
Dutch Safety Board	Interview report	−	−	−	−	−	−	−	−	−	−	−	−	−
RR1.1 Rapid response actions exist in cases of unexplained death														
RR1.2 It has been defined who will lead the investigation to determine the cause of death														
RR1.3 It has been defined which professionals have to be involved in the investigation to determine the cause of death														
RR1.4 It has been defined what has to be investigated. This includes: data collection from relevant institutions and professionals, postmortem examination and investigation at the place of death and circumstances of the death														
RR1.5 Results are collected and represented according to national criteria														
RR1.6 It has been defined how the collaboration between physicians and the municipal forensic physician could be constituted														
RR1.7 It has been defined how often and when the involved professionals have to discuss the results of the investigation to determine the cause of death														
RR1.8 Relevant institutions and professionals, such as school and GP, are consulted to get relevant information about the possible cause of death														
CDO1.1 The results of the review are passed on to a national institution														
CDO1.2 A format to get specific data about a particular cause of death is used														
CDO1.3 The actions of professionals involved in determining the cause of death are analyzed														
CDO1.4 Feedback is given to professionals on their actions in determining the cause of death														
CDO1.5 New relevant information regarding the cause of death and factors contributing to the death, which is obtained in the long run, is provided to all professionals involved around the death														

## Appendix 2

**Table 4:** Extent to which procedures of Dutch organizations covered the CDR objective *‘Identify avoidable factors that give directions for prevention’* (yes = +; to a limited extent = ±; no = −)

Organization/professional	Title of document available for analysis	RR2.1	RR2.2	CDO2.1	CDO2.2	CDO2.3	CDO2.4
Department of Pediatrics and GP	Dutch Association for Pediatrics-Action protocol after cot death	−	+	+	−	+	−
	Death of a Child	−	−	−	−	−	−
	Emergency Baptism	−	−	−	−	−	−
	Deceasing or Dying	−	−	−	−	−	−
	Procedures in External Cause of Death	−	−	−	−	−	−
Forensic Medical Service	Work Instruction ‘Reporting Deceased Minors’	−	−	−	−	−	−
	Guideline Forensic Postmortem Examination	±	−	−	−	−	−
Ambulance Service	National Protocol Ambulance Care	−	−	−	−	−	−
Preventive Child Healthcare/Municipal Health Services	Guideline Counseling Families in Child Death	−	−	−	−	−	−
	Protocol Large-scale Sexual Abuse	±	−	−	−	−	−
Hospital Social worker	Interview report	−	−	−	−	±	−
Mental health trust	Suicide and External Cause of Death	−	−	±	−	−	−
	External Cause of Death inside of the Clinic	−	−	−	−	−	−
	External Cause of Death outside of the Clinic	−	−	−	−	−	−
	External Cause of Death in Ambulatory Patient Outside of the Clinic	−	−	−	−	−	−
MEE	Interview report	−	−	−	−	−	−
Child Welfare Agency	Guidelines Death of a Juvenile Client	−	−	−	−	−	−
Child Protection Service	Interview report	+	+	+	±	+	±
Police	Interview report	−	−	±	−	−	−
Public Prosecutor	Interview report	±	±	±	−	±	±
School/daycare/playgroup	Protocol in Case of Death	−	−	−	−	−	−
Perined	Perined	±	−	+	±	+	+
National Cot Death Study Group	Dutch Cot Death Foundation	±	±	+	−	+	+
Dutch Cot Death Foundation	Interview report	+	+	+	−	+	+
Dutch Safety First Association	Interview report	−	−	±	−	±	±
Consumer Safety Institute	Interview report	±	−	±	±	±	±
Dutch Safety Board	Interview report	+	−	±	±	±	+
RR2.1 Relevant institutions and professionals, such as school and GP, are consulted to get more information about the child, his/her social circumstances and environment in the context of avoidable factors of child mortality							
RR2.2 During data collection from relevant institutions and professionals, postmortem examination and investigation at the place of death and circumstances of the death, attention is paid to (new) avoidable factors of child mortality							
CDO2.1 Avoidable factors of child mortality and lessons learned are identified							
CDO2.2 A distinction is made in factors intrinsic to the child, family and environmental factors, parenting capacity and service provision							
CDO2.3 Professionals involved work together with regional and national institutions to identify lessons learned							
CDO2.4 After identifying avoidable factors of child mortality, the extent of the problem is determined and (groups of) people most affected by the problem are sorted out							

## Appendix 3

**Table 5:** Extent to which procedures of Dutch organizations covered CDR objective ‘Translate the results into possible interventions’ (yes = +; to a limited extent = ±; no = −)

Organization/professional	Title of document available for analysis	RR3.1	RR3.2	CDO3.1	CDO3.2	CDO3.3	CDO3.4
Department of Pediatrics and GP	Dutch Association for Pediatrics-Actionprotocol after cot death	−	−	−	−	−	−
	Death of a Child	−	−	−	−	−	−
	Emergency Baptism	−	−	−	−	−	−
	Deceasing or Dying	−	−	−	−	−	−
	Procedures in External Cause of Death	−	−	−	−	−	−
Forensic Medical Service	Work Instruction ‘Reporting Deceased Minors’	−	−	−	−	−	−
	Guideline Forensic Postmortem Examination	−	−	−	−	−	−
Ambulance Service	National Protocol Ambulance Care	±	−	−	−	−	−
Preventive Child Healthcare/Municipal Health Services	Guideline Counseling Families in Child Death	−	−	−	−	−	−
	Protocol Large scale Sexual Abuse	+	−	−	±	−	−
Hospital Social worker	Interview report	−	−	−	−	−	−
Mental health trust	Suicide and External Cause of Death	−	−	−	±	−	−
	External Cause of Death inside of the Clinic	+	+	±	+	−	±
	External Cause of Death outside of the Clinic	+	+	±	+	−	±
	External Cause of Death in Ambulatory Patient Outside of the Clinic	−	−	−	±	−	−
MEE	Interview report	−	−	−	−	−	−
Child Welfare Agency	Guidelines Death of a Juvenile Client	+	±	−	−	−	−
Child Protection Service	Interview report	+	+	±	+	±	−
Police	Interview report	±	−	−	−	−	−
Public Prosecutor	Interview report	−	−	−	±	±	−
School	Protocol in Case of Death	−	−	−	−	−	−
Perined	Perined	−	−	+	+	±	+
National Cot Death Study Group	Dutch Cot Death Foundation	−	−	+	+	+	−
Dutch Cot Death Foundation	Interview report	−	−	+	+	+	−
Dutch Safety First Association	Interview report	−	−	−	+	+	+
Consumer Safety Institute	Interview report	−	−	−	+	+	+
Dutch Safety Board	Interview report	−	−	±	+	+	+
RR3.1 Information relevant for immediate prevention (e.g., protection of other children in the family) is discussed by the rapid response team							
RR3.2 It has been defined which immediate preventive measures have to be taken, when necessary							
CDO3.1 Research ends with a discussion of how such a death can be avoided in the future							
CDO3.2 Recommendations, actions to be performed and lessons learned are passed on to relevant authorities or individuals							
CDO3.3 Recommendations, actions to be performed and lessons learned are passed on to governmental institutions to improve public health							
CDO3.4 It has been defined who is responsible for (taking care of) carrying out the improvements							

## Appendix 4

**Table 6:** Extent to which procedures of Dutch organizations covered the CDR objective *‘Support to the family’* (yes = +, to a limited extent = ±, no = −)

Organization/professional	Title of document available for analysis	RR4.1	RR4.2	RR4.3	RR4.4	RR4.5	RR4.6	CDO4.1	CDO4.2	CDO4.3
Department of Pediatrics and GP	Dutch Association for Pediatrics-Action Protocol after Cot Death	±	−	−	+	−	−	−	±	−
	Death of a Child	+	+	+	−	−	+	+	+	−
	Emergency Baptism	±	−	−	−	−	−	−	−	±
	Deceasing or Dying	+	±	+	±	−	−	−	−	−
	Procedures in External Cause of Death	−	−	−	−	−	−	−	−	−
Forensic Medical Service	Work Instruction ‘Reporting Deceased Minors’	−	−	−	−	−	−	−	−	−
	Guideline Forensic Postmortem Examination	−	−	−	−	−	−	−	−	−
Ambulance Service	National Protocol Ambulance Care	−	−	−	−	−	−	−	−	−
Preventive Child Healthcare-Municipal Health Services	Guideline Counseling Families in Child Death	+	−	−	−	−	+	−	±	+
	Protocol Large scale Sexual Abuse	+	±	−	+	−	+	+	+	+
Hospital social worker	Interview report	+	+	−	−	−	+	±	±	+
Mental health trust	Suicide and External Cause of Death	−	−	−	−	−	+	±	−	±
	External Cause of Death inside of the Clinic	±	−	−	±	−	+	−	−	−
	External Cause of Death outside of the Clinic	±	−	−	±	−	+	−	−	−
	External Cause of Death in Ambulatory Patient Outside of the Clinic	−	−	−	−	−	+	−	−	−
MEE	Interview report	−	−	−	−	−	−	−	−	−
Child Welfare Agency	Guidelines Death of a Juvenile Client	+	−	−	−	−	±	−	−	−
Child Protection Service	Interview report	−	−	−	−	−	−	−	±	−
Police	Interview report	−	−	−	+	−	−	−	−	−
Public Prosecutor	Interview report	±	−	−	−	−	−	−	−	−
School	Protocol in Case of Death	+	−	−	−	−	±	−	±	−
Perined	Perined	−	−	−	−	−	−	−	±	−
National Cot Death Study Group	Dutch Cot Death Foundation	±	−	−	−	−	−	−	+	−
Dutch Cot Death Foundation	Interview report	±	−	−	−	−	+	−	+	±
Dutch Safety First Association	Interview report	−	−	−	−	−	−	−	−	−
Consumer Safety Institute	Interview report	−	−	−	−	−	−	−	−	−
Dutch Safety Board	Interview report	−	−	−	−	−	−	−	−	−
RR4.1 The potential needs of relatives are identified										
RR4.2 When a child died in the hospital, parents are supported by a designated professional of the hospital										
RR4.3 When conditions permit, parents get the opportunity to be alone with their deceased child to take leave of their child										
RR4.4 Parents are informed about up-to-date findings of the investigation, unless this obstructs the investigation										
RR4.5 It has been defined how to act when parents and the deceased child do not live in the same country										
RR4.6 After completion of the rapid response, further (psychological) assistance is rendered to the relatives										
CDO4.1 The actions of professionals in supporting grief counseling to relatives are analyzed										
CDO4.2 Relatives are kept in touch in the long run, whereby feedback is given about the circumstances of and factors contributed to the death and grief counseling										
CDO4.3 The given support to relatives is monitored										
